# MiTF/TFE Translocation Renal Cell Carcinomas: From Clinical Entities to Molecular Insights

**DOI:** 10.3390/ijms23147649

**Published:** 2022-07-11

**Authors:** Audrey Simonaggio, Damien Ambrosetti, Virginie Verkarre, Marie Auvray, Stéphane Oudard, Yann-Alexandre Vano

**Affiliations:** 1 Department of Medical Oncology, Hôpital Européen Georges Pompidou, Institut du Cancer Paris CARPEM, AP-HP. Centre—Université Paris-Cité, F-75015 Paris, France; audrey.simonaggio@aphp.fr (A.S.); marie.auvray2@aphp.fr (M.A.); stephane.oudard@aphp.fr (S.O.); 2 Department of Pathology, CHU Nice, Université Côte d’Azur, F-06107 Nice, France; ambrosetti.d@chu-nice.fr; 3 Institute for Research on Cancer and Aging of Nice (IRCAN), CNRS UMR 7284, INSERM U1081, University Côte d’Azur, F-06107 Nice, France; 4 Department of Pathology, Hôpital Européen Georges Pompidou, Institut du Cancer Paris CARPEM, AP-HP. Centre—Université Paris-Cité, F-75015 Paris, France; virginie.verkarre@aphp.fr; 5 INSERM UMR-970, PARCC, Université Paris-Cité, F-75015 Paris, France; 6 Centre de Recherche des Cordeliers, INSERM, Université Paris-Cité, Sorbonne Université, F-75006 Paris, France

**Keywords:** translocation renal cell carcinomas, TFE3, TFEB, MITF

## Abstract

MiTF/TFE translocation renal cell carcinoma (tRCC) is a rare and aggressive subtype of RCC representing the most prevalent RCC in the pediatric population (up to 40%) and making up 4% of all RCCs in adults. It is characterized by translocations involving either TFE3 (TFE3-tRCC), TFEB (TFEB-tRCC) or MITF, all members of the MIT family (microphthalmia-associated transcriptional factor). TFE3-tRCC was first recognized in the World Health Organization (WHO) classification of kidney cancers in 2004. In contrast to TFEB-tRCC, TFE3-tRCC is associated with many partners that can be detected by RNA or exome sequencing. Both diagnoses of TFE3 and TFEB-tRCC are performed on morphological and immunohistochemical features, but, to date, TFE break-apart fluorescent in situ hybridization (FISH) remains the gold standard for diagnosis. The clinical behavior of tRCC is heterogeneous and more aggressive in adults. Management of metastatic tRCC is challenging, especially in the younger population, and data are scarce. Efficacy of the standard of care-targeted therapies and immune checkpoint inhibitors remains low. Recent integrative exome and RNA sequencing analyses have provided a better understanding of the biological heterogeneity, which can contribute to a better therapeutic approach. We describe the clinico-pathological entities, the response to systemic therapy and the molecular features and techniques used to diagnose tRCC.

## 1. Introduction

Kidney cancer accounts for 3 to 5% of all cancers and is the seventh most frequently diagnosed malignancy among men and the tenth among women. Each year, about 330,000 new cases are diagnosed worldwide and 120,000 patients die from kidney cancer [1]. Clear cell renal cell carcinoma (ccRCC) is the most frequent type of kidney cancer making up almost 75% of all renal cell carcinoma cases. The remaining 25% are made of non-clear cell RCCs (nccRCC) including papillary RCC (pRCC), chromophobe RCC (cRCC), MiTF/TFE translocation renal cell carcinoma (tRCC), collecting duct and other rare subtypes. About 5% of tumors remain unclassified [2].

MiTF/TFE translocation renal cell carcinomas represent up to 40% of all pediatric and adolescent RCCs [3] and 1–4% of adult RCCs [4]. Because of the morphological overlap with more common RCCs, the percentage of tRCCs in adults is probably underestimated if specific immunohistochemistry is not performed. The Xp11 translocation RCCs harbor gene fusions involving *TFE3*, whereas t(6;11) RCCs harbor gene fusions involving *TFEB*. The t(6;11) RCCs are less common than the Xp11 ones and account for 0.02% of all renal carcinomas [5].

The Xp11 RCCs (TFE3-tRCC) were first described in 1991 by Tomlinson et al. in a 17-month-old child and considered as “juvenile RCC” [6]. TFE3-tRCC represents 89% of all tRCCs [7]. Although TFE3-tRCC predominantly affects children and young adults, it can also be encountered in older populations. In 2004, TFE3-tRCC was recognized as a specific entity in the World Health Organization’s (WHO’s) kidney cancers [8].

The t(6;11) RCCs were identified later in 2001 by Argani et al. in children and introduced in the 2013 International Society of Urologic Pathology (ISUP) Vancouver classification [5,9]. According to a recent comprehensive review article by Argani et al. fewer than 100 cases of TFEB CRC have been reported in the literature worldwide [10]. Subsequently, TFE3 and TFEB were grouped together under the title “MITF/TFE translocation renal carcinoma” in the 2016 WHO classification [11] and then were separated into two distinct molecularly-defined entities in the recent WHO classification, 2022, as TFE3-rearranged RCC and TFEB-rearranged RCC [12]. Fusions involving *MITF* are very rare and have been identified in only 2% of cases [7].

## 2. Clinical Entities

In a recent review considering 403 confirmed TFE3-tRCCs, Calio et al. reported a female predominance (F–M ratio, 1.6:1) and an average age of onset of 40 (most frequent decade age group: 20–29) [13]. A meta-analysis of observational studies led by Cheng et al. also confirmed this female predominance with a pooled OR of 3.93 (95% CI: 1.66–9.34) without a difference between men and women for the incidence of distant metastases, tumor stage or overall survival [14]. One-third of all tumors were asymptomatic at the time of diagnosis. In contrast to other RCCs, smoking, obesity and hypertension were not reported as risk factors. Argani et al. reported an association in children between a history of exposure to cytotoxic chemotherapy and the development of tRCC, concerning six patients (15%) of a total of 39 confirmed tRCCs [5,13,15].

In a recent review considering 403 confirmed TFE3-tRCCs, Calio et al. reported a female predominance (F–M ratio, 1.6:1) and an average age of onset of 40 (most frequent decade age group: 20–29) [13]. A meta-analysis of observational studies led by Cheng et al. also confirmed this female predominance with a pooled OR of 3.93 (95% CI: 1.66–9.34) without a difference between men and women for the incidence of distant metastases, tumor stage or overall survival [14]. One-third of all tumors were asymptomatic at the time of diagnosis. In contrast to other RCCs, smoking, obesity and hypertension were not reported as risk factors. Argani et al. reported an association in children between a history of exposure to cytotoxic chemotherapy and the development of tRCC, concerning six patients (15%) of a total of 39 confirmed tRCCs [5,13,15].

The prognosis of TFE3-tRCC varies from an indolent disease to a highly aggressive disease, depending in part on the age of the patient at the time of diagnosis. The prognosis appears favorable with a more indolent tumor for children up to 16 years old. Considering the possible occurrence of late metastasis at 20 to 30 years of age, a long-term follow-up is required in TFE3-tRCC [16]. Patients with lymph node involvement (N+) but without metastatic spread (M0) maintained a favorable prognosis following surgery. In a small cohort study led by Wang et al. the estimated 5-year overall survival rate and progression-free survival rate were, respectively, 86.6% and 70.3% for localized disease [17]. Adult patients most often present with advanced and aggressive metastatic disease, with a median overall survival after diagnosis of approximately 18 months [18]. Some fusion partners such as ASPL/ASPSCR1-TFE3 fusion have been associated with having worse outcomes [19,20].

Approximately 60 cases of TFEB-tRCC are described in the literature, occurring mainly in children and adolescents and young adults. The mean age of onset is 34 years (most frequent decade age group: 30–39) with no gender predominance identified [13]. The main TFE3 and TFEB tRCC and ccRCC clinical features are summarized in Table 1.

## 3. Pathological Features of TFE-tRCC

Both TFE3 and TFEB tRCCs may overlap with each other’s and with other RCCs’ morphologies, in particular with clear cell RCC, papillary RCC or, more rarely, clear cell papillary renal cell carcinomas as well as perivascular epithelioid cell tumors (PEComas). These overlapped features may lead to the misclassification of tRCC if the specific immunohistochemistry required for diagnosis is missing, as observed, in particular, in TCGA RCC cohorts [7]. TFE3 tRCC diagnosis is proposed based on the analysis of both morphological and immunohistochemical features. The minimal panel of antibodies contains carbonic anhydrase-IX (CAIX), TFE3 and PAX8, and melanocytic markers such as HMB-45 and Melan-A, AMACR, cytokeratin 7 (CK-7), pancytokeratin or epithelial membrane antigen (EMA). Cathepsin K could also be helpful. Immunohistochemical features between MiT family tRCCs and their main differential diagnoses are summarized in Table 2. However, diagnoses have to be confirmed by molecular analysis as detailed below.

**Figure 1:**
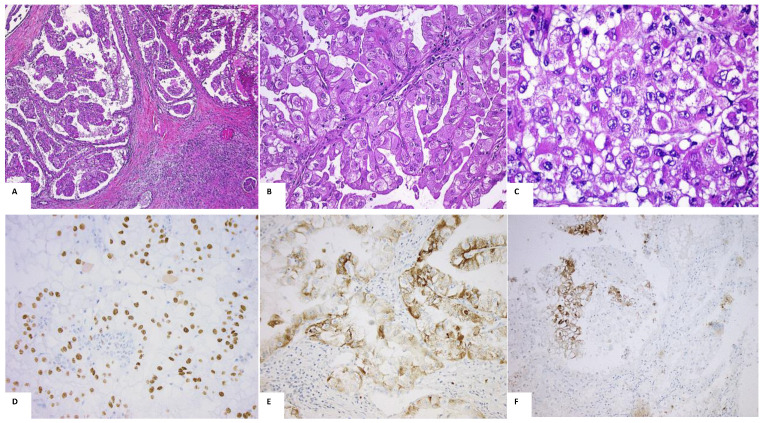
Morphological and immunohistochemical features of TFE3-tRCC. Captions: (**A**–**C**): Morphology and features. (**A**/**B**) Papillary architecture; C: eosinophilic cells with prominent nucleoli (HES staining, magnification (**A**) ×50, (**B**) ×200, (**C**) ×200); (**D**–**F**): immunohistochemical features. (**D**): TFE3 positivity of the majority of nuclei; (**E**): p504 cytoplasmic positivity for some cells; (**F**): CAIX positivity in hypoxic territory (TFE3/p504/CAIX IHC, respectively, magnification ×200); *tRCC: translocation renal cell carcinoma; HES: hematoxylin–eosin–safran*.

Microscopic typical features of TFE3-tRCC harbor a papillary architecture, mostly composed of large epithelioid cells with either clear or eosinophilic cytoplasms (Figure 1A–C) and contain frequent psammoma calcifications. However, psammoma calcifications may be absent or scarce and several other morphologies can be seen, ranging from compacted nested and trabecular or with a more cystic appearance and cells with smaller cytoplasms. Nuclear features are not specific but could contain nuclear pseudo inclusions, subnuclear clearing and linear nuclear arrays, and could more frequently harbor high WHO/ISUP nucleolar grade ≥3 [23]. A few morphological features have been identified more frequently in some fusion partners such as the presence of subnuclear vacuoles in SFPQ_TFE3, NONO-TFE3 or RBM10-TFE3 [10] (Figure 1).

Immunohistochemical features of TFE3-tRCC are characterized by a specific strong nuclear expression of TFE3 (antibody targeting the c-terminal portion of TFE3) sometimes associated with positive cathepsin K (approximately 60% of cases) (Figure 1). Scattered positive melanocytic markers (HMB-45 or Melan-A) could be seen as in TFEB-tRCC [10]. Expression of PAX8, a common biomarker to all renal tumors originated from a renal tubular origin is conserved in most cases of TFE3-tRCC in contrast to pancytokeratin or EMA that is often under expressed. In contrast to clear cell RCCs and papillary RCCs, respectively, CAIX (carbonic anhydrase-IX) is usually negative or only focal, and CK7 is rarely diffused [10,24,25].

**Figure 2:**
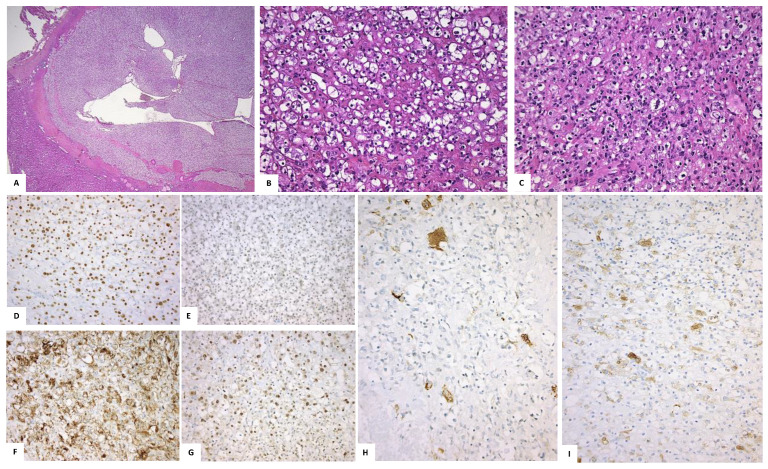
Morphological and immunohistochemical features of TFEB-tRCC mimicking clear cell RCC. (**A**–**C**): Morphology and features. (**A**,**B**): Massive architecture; (**B**,**C**): mixed eosinophilic and clarified cells (HES staining, magnification (**A**) ×12.5, (**B**,**C**) ×200); (**D**–**F**): immunohistochemical features. (**D**): PAX8 positivity; (**E**): CAIX negativity; (**F**): AE1–AE3 positivity for some cells; (**G**): TFE3 nuclei positivity for some cells; (**H**): HMB45 cytoplasmic positivity for few cells; (**I**): Melan-A cytoplasmic positivity for few cells (**D**): PAX8, (**E**): CAIX, (**F**): AE1-AE3, (**G**): TFE3, (**H**): HMB45, (**I**): Melan-A IHC, respectively, magnification ×200); tRCC: translocation renal cell carcinoma; HES: hematoxylin–eosin–safran.

TFEB-tRCC typically demonstrates a dual proliferation organized in the papillary architecture comprising larger epithelioid cells and smaller cells clustered around basement membrane material. Tumoral cells could be either clear or eosinophilic (Figure 2). TFEB-tRCC expresses Melan-A, cathepsin K and HMB45 (almost focally) but these are usually negative or only focally positive for epithelial markers (Figure 2). In contrast to PEComas, they usually express PAX8 [26]. To date, the TFEB antibody has not been used in routine practice for diagnosis.

## 4. Molecular Features

### 4.1. Molecular Tool for tRCC Diagnosis

**Figure 3:**
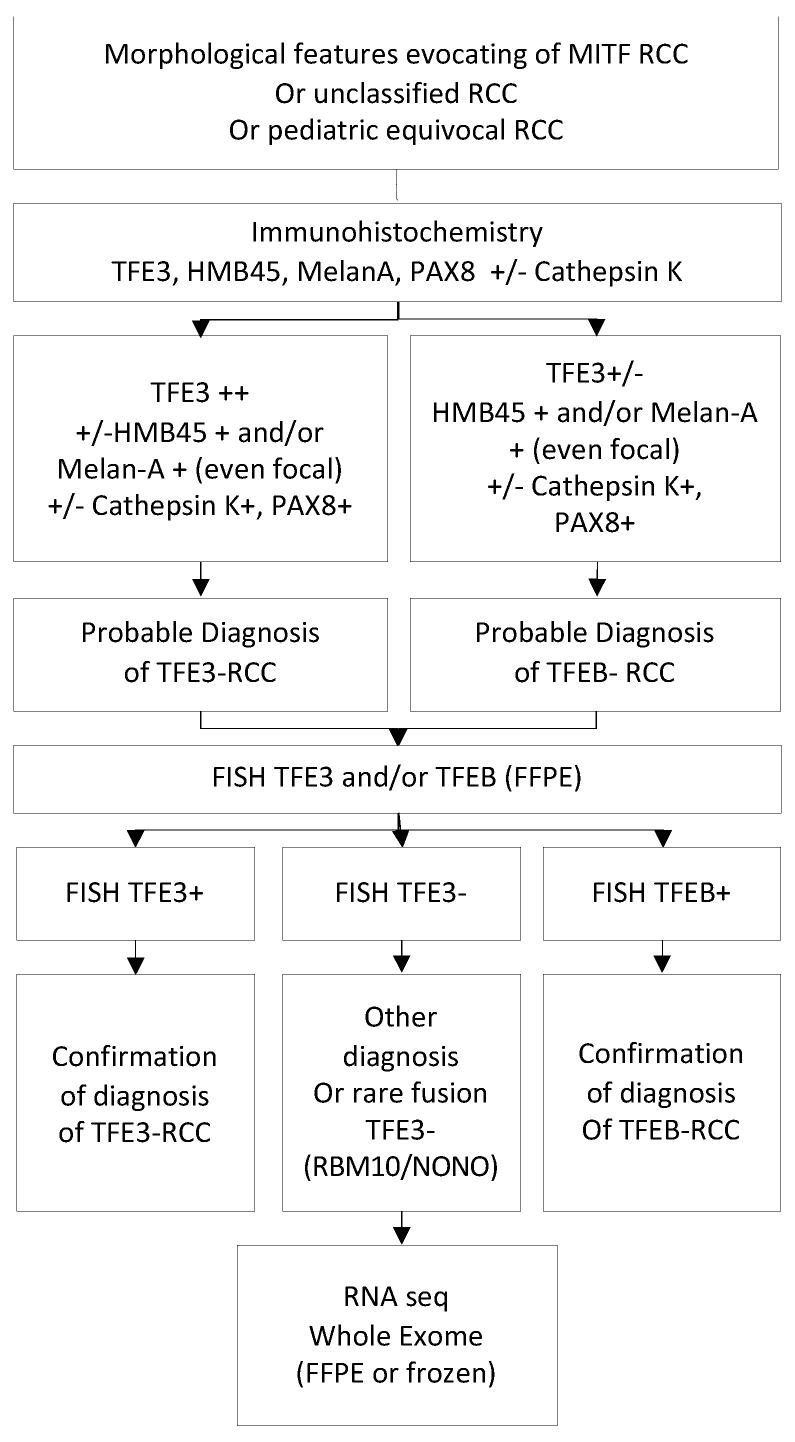
Proposed algorithm of pathologic analyses to identify tRCC; MITF: microphthalmia-associated transcription factor; RCC: translocation renal cell carcinoma; FISH: fluorescence in situ hybridization.

The historical conventional karyotype was replaced several years ago by molecular cytogenetic or genetic analysis; the aim of these is to confirm the pathological suspected diagnosis. Different methods can be used to confirm the diagnosis, depending in practice on the local availability. We propose an algorithm for the diagnosis of tRCC in Figure 3.

Break-apart TFE3 or TFEB fluorescence in situ hybridization (FISH) performed on formalin-fixed and paraffin-embedded (FFPE) tissue sections is the most widespread method used and has long been considered the gold standard. This technique allows confirmation of the TFE3 or TFEB rearrangement without providing information on the fusion partner. Typical break-apart signals of TFE3 gene translocation consist of split red and green signals, the normal result being a merged hybridization signal, as for TFEB. FISH assays may show equivocal or false-negative results due to intrachromosomal inversions involving TFE3, resulting in a subtly spaced fluorescence signal that is less obvious to visualize than a translocation. This difficulty has been demonstrated for rearrangements involving NONO, *GRIPAP1, RBMX* and RBM10, consisting of the pericentric or paracentric inversion of chromosome X, respectively, for which there is close proximity of the genes involved in the rearrangement [27,28]. In the case of a strong suspicion of tRCC with negative FISH, specific targeted RT-PCR or RNA sequencing (RNA-seq), or whole exome sequencing is recommended. 

A new sensitive and specific biomarker of tRCC (TRIM63) that has emerged from RNA-seq analysis has recently been tested by in situ hybridization RNA (RNA-ISH). The expression of TRIM63 by RNA-ISH was highly expressed in all cases of TFE3/TFEB-tRCCs compared to other RCCs and was also positive in TFE3 FISH false-negatives associated with RBM10-TFE3 inversion [29].

A complementary molecular diagnosis can also be performed by RNA-seq analysis either by targeted sequencing or a fusion panel. The increasing use of the RNA-seq has allowed the identification of many new transcript and gene fusion partners [30]. Taking advantage of recent advances in the prognostic significance of certain fusion partners, this powerful technique could become the future gold standard for diagnosis. Whole exome sequencing can also be used to identify fusion partners with the advantage of providing other interesting information regarding copy number variation or variation in other genes that may impact patient outcomes or predict treatment efficacy [20,30].

### 4.2. Partners of Fusion with TFE3

TFE3 is a family member of MITF transcription factors. It induces the expression of proto-oncogene coding for a tyrosine kinase receptor whose activation triggers downstream signaling pathways responsible for unregulated cell proliferation. Initially described in 1986, XP11 tRCC is defined by the fusion between the TFE3 transcription factor gene (locus Xp11.2) and different partner genes. Approximately 20 distinct partners have been described to date [10,20] (Table 3). The most common partners involved in TFE3-tRCC are *PRCC* (papillary renal cell carcinoma) with t(X;1)(p11.2;q21.2), *ASPSCR1*(*ASPL)* (alveolar soft part sarcoma locus) with the t(X;17)(p11.2;q25) and *SFPQ* (splicing factor proline- and glutamine-rich protein) with t(X;1)(p11.2;p34) [7,20,31]. Less recurrent *TFE3* fusion partners include *NONO* (non-POU domain containing octamer binding) resulting from inv(X) (p11.2q12) [32], *CLTC* (clathrin heavy chain) resulting from t(X; 17) (p11.2; q23) [33] and *RBM10* (RNA binding motif protein 10) resulting from inv(X) (p11.2p11.23) [34]. Despite great diversity breakpoints of TFE3, all fusions preserved the C-terminal helix–loop–helix/leucine zipper domain (HLH-LZ) of the MiT/TFE transcription factor, which is critical for dimerization and DNA binding, and thus activation [7].

### 4.3. Additional Comprehensive Molecular Features of TFE3-tRCC

In addition to previous molecular studies, recent integrative analyses performed on larger cohorts have considerably improved our understanding of the molecular landscape of TFE3-tRCC and highlighted its heterogeneity [7,20].

Beyond translocations, additional quantitative genomic alterations have also been found in cases of tRCC [45]. However, copy number variation (CNV) assessed by whole-exome sequencing (WES) or comparative genomic hybridization has a significantly lower burden compared to ccRCC, pRCC and chRCC [7,20]. tRCC tumors from young patients ( <18 years) display fewer genetic alterations compared to tumors from adult patients [45]. TFE3-tRCC can be associated with the hemizygous loss of chromosome 3p (29–31%), 17p (31%), 9p (23–41%), chromosome 18 (29.4%) and chromosome 22q (18.8%), as well as a gain of 17q (20–44%) [7,45,46]. Combining data of two cohorts (MSK-impact and TCGA cohort), a statistically significant association between copy number-aberrant tumor status and poor survival in tRCC was identified [46]. Some chromosome alterations have been associated with a worse outcome such as the gain of 17q or loss of 22q [20,45]. 

The rate of tumor mutational burden (TMB) is significantly lower in tRCC (median 0.82 (0.43–1.28) per megabase) on WES, compared to those in ccRCC and pRCC and comparable to chRCC [7]. Pediatric tRCC contains a lower TMB compared to that in adults [7,20,46]. CDKN2A/2B are the most often altered genes in tRCCs (19%), notably due to deletion at locus (9p21.3). Of the most frequently mutated genes in tRCCs, none exceeded a frequency of 10%, and this included genes involved in the DNA-damage response repair (DDR) (22.7%) (*ATM, BRCA2* and *WRN*), genes involved in ATP-dependent chromatin remodeling via the switch/sucrose non-fermentable (SWI/SNF) (68.2%) complex (*ARID1A* and SMARCA4) and mutations in *TERT* (6.8%; primarily non-coding mutations in the TERT promoter) [7,46]. *TERT* promoter mutations were found exclusively in high-stage tumors, suggesting an association with molecular progression in tRCCs. An increased frequency of oncogenic events (including somatic mutations and CNVs) in adult patients could presumably be driving more aggressive disease phenotypes [46]. Notably, mutations in genes that are significantly mutated in ccRCC, such as VHL, tended to be depleted in tRCCs [7]. In particular, the odds ratio of VHL mutation frequency between tRCCs and ccRCCs was less than 0.05 in the comprehensive multi-omics study by Bakouny et al. [7].

### 4.4. Transcriptomic Signatures of TFE3-tRCC

Among a few RNA-seq studies performed on tRCCs, distinct transcriptomic signatures have been identified compared with other tumors or RCCs [20]. The angiogenesis gene expression signature was higher in tRCCs compared to papillary RCCs and lower but almost equivalent with respect to ccRCCs [46]. An enrichment of transcriptional pathways involving proliferation (E2F targets and G2M checkpoint), PI3K/ATK/mTOR and p53 signaling have also been described in TFE3-tRCC [20]. Compared to all tumor types, ASPSCR1-TFE3, NONO-TFE3, PRCC-TFE3 and SFPQ-TFE3 signatures are characterized by the overexpression of genes implicated in mTORC1 signaling, the antioxidant stress response, ROS sensing and the response to oxidative stress and xenobiotics including activation of the NRF2 pathway [7]. In tRCCs, the overexpression of PD-L1 on RNA-Seq is consistent with the findings from a large study performed on the immunohistochemistry in which tRCCs showed PD-L1 overexpression in both tumor-infiltrating mononuclear cells (90%) and tumoral cells (30%) [46,47]. Compared with TCGA RCC subtypes (TCGA database), TFE3-tRCC has an increased signature in the T helper 2 cell and natural killer cell, while having a decreased signature in the activated dendritic cell and plasmacytoid dendritic cell. CD8 + T cell, T cell and macrophage signatures were also lower relative to ccRCC, which was also demonstrated by immunohistochemistry (low CD8 + T cell infiltrations, immunohistochemistry confirmed in two-thirds (74.6%, 47/63) of tRCC) [20]. However, transcriptomic profiles were heterogeneous among 54 cases studied with TFE3 that allowed the identification and characterization of five molecular subtypes of TFE3-tRCC with distinct representative genes involving stroma, angiogenesis, proliferation and KRAS down [20]. In particular, all tumors with ASPSCR1-TFE3 fusion, which have been classified into the high angiogenesis/stroma/proliferation cluster, exhibited worse survival [20].

### 4.5. Molecular Features of TFEB-tRCC

TFEB (6p21) is the second gene that encodes the transcription factor MITF and has been described as less frequently rearranged in RCC. These rearrangements induce high expressions of the TFEB protein. As described for TFE3-tRCC, microscopic morphology, immunohistochemistry and the integration of genetic data are necessary for an accurate diagnosis. Most cases of TFEB translocation reported so far had the Malat1/Alpha gene located at 11q12 as a partner. More recent and rarer cases have been described partnered with ACTB, NEAT1 [48], COL21A1 and CADM2 [49]. As with TFE3 tRCCs, the easiest way to confirm the TFEB tRCC diagnosis is the single break-apart TFEB FISH [26]. Genomic amplification, which is very rare in RCCs, has been reported for TFEB [23] and included in the “TFEB-rearranged RCC” group in the 2022 WHO classification [12]. These tumors are mainly high grade and quite distinct from TFEB-tRCC in a clinical, pathological and genetic point of view. Thus, TFEB-amplified tRCC may be considered as a different subtype.

## 5. Response to Modern Systemic Therapies

In the metastatic setting, no dedicated prospective trial has been reported for tRCCs. As with other non-clear cell entities, most metastatic tRCCs are treated with tyrosine kinase inhibitors targeting VEGFR (VEGFR-TKI). The main available data (from retrospective studies) related to tRCCs′ response to systemic therapies are summarized in Table 4.

In 2010, Choueiri et al. reported the survival outcomes of 15 Xp11.2 adult patients harboring a strong TFE-3 nuclear immunostaining and receiving VEGF-targeted therapy (ten sunitinib, three sorafenib, one bevacizumab and one ramucirumab) [50]. Eighty percent of patients were female and the median age was 41 years. One-third of patients had received prior systemic therapy, and two-thirds were in a first-line setting. With a median follow-up of 19 months, median progression-free survival (PFS) and overall survival (OS) were, respectively, 7.1 months (95% CI: 1.7–27) and 14.3 months (95% CI 2.7-NR). By RECIST, three patients achieved a partial response (durations of response were, respectively, 7, 13 and 27 mon), seven achieved stable disease and five had a progressive disease. Despite the potential bias inherent to this retrospective and small sample size study, it demonstrated that VEGF-targeted therapy can be of benefit for Xp11.2 adult patients. The same year, Malouf et al. [51] reported the results of a French retrospective study focusing on 21 Xp11.2 adult and young patients with metastatic and evaluable disease treated in a first-line setting. In the first-line setting, 11 patients received sunitinib and nine received cytokines (IFN-alpha and IL-2 or high-dose IL-2). Four (36%) objective responses (one complete and three partial responses) were achieved with sunitinib and only one (11%) partial response with cytokines. Median PFS and OS with sunitinib were 8.2 months and not reached, and were 2 and 17 months with cytokines, respectively. As in conventional metastatic RCC, sunitinib appeared more effective than cytokines. It should be noted that these earlier studies used TFE3 staining to confirm the diagnosis, potentially including patients who did not have a genomic translocation. Interestingly, activation of the NRF2 pathway, which has recently been identified as a hallmark of tRCC, has previously been shown to be associated with resistance to several targeted therapies used in the treatment of RCC, including VEGFR-TKIs [7].

Translocation RCCs are known to strongly express MET. Thus, cabozantinib, a VEGFR-TKI that also inhibits MET and AXL, could be a promising treatment. Thouvenin et al. reported at the 2021 ASCO Genito Urinary Congress the results of a multicenter, retrospective, international cohort study focusing on tRCC patients treated with cabozantinib regardless of the line of treatment [54]. Twenty-four patients (21 with TFE3 and 3 with TFEB translocations) were evaluable for response and included in the study. Among them, the objective response rate was 17%, and the median PFS and OS were 8.4 and 17 months, respectively. With a disease control rate (objective response plus stable disease) of 62.5%, cabozantinib may be considered as a new therapeutic option for tRCCs. Prospective and larger studies are required to confirm these results.

PD-L1 expression by tumor cells and tumor-infiltrating mononuclear cells was reported in 30% and 90% of tRCC cases, respectively, justifying the analysis of the benefits of ICIs in these population [47]. In addition, Bakouny et al. found that tRCCs harbor a higher percentage of CD8+PD1+TIM3-LAG3+ T cells compared to ccRCCs. They concluded that tRCCs may benefit from ICI as a result of a permissive immune microenvironment [7]. A recent multicenter retrospective study enrolled 24 tRCC patients (21 TFE3 and 4 TFEB) treated with ICI in a second-line line setting or later [52]. Of the 24 patients, 17 received nivolumab, 3 received ipilimumab and 4 received an ICI-based combination. The median PFS was 2.5 months (range: 1–40). By RECIST 1.1, four patients (16.6%) experienced a partial response and three experienced stable disease (12.5%). Interestingly, tumor genomics was available in eight patients. The median tumor mutation load was lower than that of the ccRCCs from the TCGA dataset. Two patients with clinical benefits harbored the mutation of bromodomain member genes (PBRM1 and BRD8) consistent with a previous report on ccRCCs [55].

In parallel with the evolution of the standard of care for first-line metastatic ccRCCs [56], a number of combinations including anti-PD-(L)1 antibody, with VEGFR-TKI or with anti-CTLA-4 antibody, are being investigated in nccRCCs, including tRCCs. The main ongoing trials are listed in Table 5.

## 6. Conclusions

Translocation RCCs are rare entities associated with those of younger age at onset and a more aggressive disease than in classical ccRCCs. Because of the rarity of this family of renal cell carcinomas, no specific prospective trials have been published to date and metastatic patients are treated with the same molecules as those with ccRCC, albeit with lower efficacy. Clear progress has been made in the diagnosis and the molecular identification of translocation partners. Nevertheless, these advances have not yet translated into major advances in the management of metastatic disease. First, it is likely that the incidence of these forms in adults >45 years old is underestimated due to the absence of systematic research of the fusion transcript in this age category. As the prognosis is poorer for the same stage than for ccRCC, the precise identification of these rare variants seems crucial. Immunohistochemical analysis using the recommended minimum antibody panel (CAIX, TFE3, PAX8, HMB-45 and Melan-A, Cathepsin K, AMACR, CK-7, EMA) could be advised routinely for every case of RCC, regardless of the patient′s age and the microscopic features of the tumor. In addition, the development of technologies such as RNA-seq allow further progress to be made in identifying deregulated signaling pathways and in the composition of the microenvironment. Thus, we learned that the microenvironment of tRCC is even more immunopermissive than that of ccRCC. However, the limited clinical data on the efficacy of anti-PD-1 alone are disappointing. Efficacy results from new immune-based combinations in nccRCC will be reported in the next months or years. Nevertheless, we can anticipate that the limited number of patients with tRCC in these trials will make the results difficult to interpret. More in-depth molecular characterization including transcriptomic pathway-specific signatures such as angiogenic, immune or metabolic ones, is needed to bridge the gap between disease understanding and effective systemic treatment.

## Figures and Tables

**Table 1:** Comparison between ccRCC and tRCC (TFE3, TFEB) patients.

	ccRCC [21,22]	TFE3 RCC [3,7,13]	TFEB RCC [4,7,13]
**Incidence**	Up to 75% of all RCCs	1–4% of all adult RCCs Up to 40% of pediatric RCCs	<0.1% of all adult RCCs
**Mean age, years**	64	40	34
**Sex ratio F–M**	2:1	1.6:1	0.75:1
**Stage at diagnosis** Metastatic	1/3 of patients	1/3 of patients Adults: 23–50% nodes involvement, mainly grade III.	Unknown
**Outcomes** Median OS (stage IV) 5-years OS rate	24 months 76% Stage I: 93%, II/III: 72%, IV: 12%	18 months (adults) 86.6% (localized) Favorable up to 16 years old. Aggressive in adult patients	Unknown Unknown
**Risk factors**	Age, sex, smoking, obesity, alcohol, hypertension, genetic predisposition (mainly Von Hippel–Lindau disease), trichloroethylene exposure	Prior chemotherapy	Unknown

OS: overall survival; tRCC: translocation renal cell carcinoma; RCC: renal cell carcinoma.

**Table 2:** Immunohistochemical features between MiT family tRCC and their main differential diagnoses.

RCC Subtypes	TFE3	Cathepsin K	HMB45	Melan-A	CAIX	CK7	AMACR
Xp11 tRCC	+	+/−	−/f+	f+/−	−/f+	−	+
t(6;11) RCC	−	+	+/−	+	−/f+	−	+
Clear cell RCC	−	−	−	−	+	−/f+	+low/−
Papillary RCC	−	−	−	−	−	+	+
Epithelioid angiomyolipoma	+	+	+	+	−	−	−

TFE3: transcription factor binding to IGHM enhancer 3; HMB45: human melanoma black45; CAIX: carbonic anhydrase-IX; CK7: cytokeratin 7; AMACR: alpha-methylacyl-CoA racemase; +: positive; f+: focally positive; −: negative; RCC: renal cell carcinoma; tRCC: translocation RCC.

**Table 3:** TFE3 fusion partners.

Gene Fusion	Chromosome Translocation	Reference
*ARID1B-TFE3*	*t(X;6)(p11.2;q25)*	[35]
*ASPSCR1-TFE3*	*t(X;17)(p11.2;q25)*	[36]
*CLTC-TFE3*	*t(X;17)(p11.2;q23)*	[33]
*DVL2-TFE3*	*t(X;17)(p11.2;p13.1)*	[31]
*FUBP1-TFE3*	*t(X;1)(p11.2;p31.1)*	[17]
*GRIPAP1-TFE3*	*inv(X)(p11.23;p11.23)*	[37]
*KAT6B*-*TFE3*	*t(X;10)(p11.2;q22.2)*	[38]
*KHSRP-TFE3*	*t(X;19)(p11.2;p13)*	[30]
*LUC7L3-TFE3*	*t(X;17)(p11.2;q21)*	[30]
*MATR3-TFE3*	*T(X;5)(p11.2;q31.2)*	[17]
*MED15-TFE3*	*t(X;22)(p11.2;q11.2)*	[17]
*NEAT1*-*TFE3*	*t(X;11)(p11.2;q13.1)*	[38]
*NonO-TFE3*	*inv(X)(p11.2;q13)*	[32]
*PARP14-TFE3*	*t(X;3)(p11.2;q23)*	[39]
*PRCC-TFE3*	*t(X;1)(p11.2;q21)*	[40]
*RBM10-TFE3*	*Inv(X)(p11.2;p11.23)*	[34]
*RBMX-TFE3*	*Inv(X)(p11;q26)*	[41]
*SETD1B-TFE3*	*t(X;12)(p11.2;q24.31)*	[42]
*SFPQ-TFE3*	*t(X;1)(p11.2;p34)*	[43]
*ZC3H4-TFE3*	*t(X;19)(p11.2;q13.32)*	[44]

**Table 4:** Main available data related to tRCCs′ response to systemic therapies.

Ref.	Type of Study	Line of Treatment	Number of Patients	Treatment	Response	Survival Outcomes
[50]	Retrospective	1.2	15 (Xp11.2, adults)	VEGF-targeted therapy (sunitinib, sorafenib, bevacizumab)	PR 3/15 SD 7/15 PD 5/15	PFS = 7.1 mon OS = 14.3 mon
[51]	Retrospective	1	20 (Xp11.2, adults, youth)	Sunitinib, cytokines	Sunitinib: CR 1/11 PR 3/11 SD 6/11 PD 1/11 Cytokines: PR 1/9 SD 2/9 PD 6/9	PFS Sunitinib: 8.2 mon Cytokines: 2 mon OS Sunitinib: not reached Cytokines: 17 mon
[51]	Retrospective	≥2	17 (Xp11.2 adults, youth)	Sunitinib, sorafenib, mTOR inhibitor	Sunitinib: PR 3/3 Sorafenib: SD 7/8 mTOR: PR 1/7, SD 6/7	PFS Sunitinib: 11 mon Sorafenib: 9 mon mTOR: 3 mon
[52]	Retrospective	≥2	24	anti-PD1, anti-CTLA4, anti-PD1–anti-CTLA4	CR 0 PR 4/24 SD 3/24	PFS = 2.5 mon OS = 24 mon
[53]	Prospective phase 2	≥1	60 (various histology,5 tRCC)	Atezolizumab +bevacizumab	ORR 20% SD 80% (tRCC)	Not reported for tRCC cohort.
[54]	Retrospective	≥1	24	Cabozantinib	CR 4% ORR = 17% DCR (ORR + SD) 62.5%	PFS = 8.4 mon OS = 17 mon

CR: complete response; DCR: disease control rate, ORR: objective response rate; OS: overall survival; PFS: progression-free survival; PD: progressive disease; PR: partial response; tRCC: translocation renal cell carcinoma; SD: stable disease.

**Table 5:** Main ongoing clinical trials enrolling translocation renal cell carcinoma patients.

Trials	Clinical Phase	Population	Number of Patients	Treatment	Primary Endpoint	Status *
KEYNOTE-B61 NCT04704219	2	nccRCC	152	Pembrolizumab +Lenvatinib (single arm)	ORR	Not yet recruiting
NCT03541902	2	nccRCC	84	Cabozantinib vs. sunitinib	PFS	Recruiting
NCT03685448	2	nccRCC	48	Cabozantinib (single arm)	ORR	Recruiting
NCT03595124	2	tRCC	40	Axitinib +nivolumab vs. Nivolumab or Axitinib	PFS	Recruiting
NCT04413123	2	nccRCC	60	Cabozantinib +nivolumab +ipilimumab	ORR	Recruiting
NCT03635892	2	nccRCC	97	Nivolumab +cabozantinib (single arm)	ORR	Recruiting
UNISoN NCT03177239/ ANZUP 1602	2	nccRCC	85	Nivolumab +ipilimumab (single arm)	ORR	Active, not recruiting
NCT03075423/ SUNNIFORECAST	2	nccRCC	306	Nivolumab +ipilimumab vs. sunitinib	OS rate at 12 mon	Recruiting

* as of 29 March 2022; DOR: duration of response; irAEs: immune-related adverse events; ORR: objective response rate; OS: overall survival; PFS: progression-free survival; tRCC: translocation renal cell carcinoma; vs.: versus.

## Data Availability

Not applicable.
